# Pigmented Villonodular Synovitis: A Retrospective Multicenter Study of 237 Cases

**DOI:** 10.1371/journal.pone.0121451

**Published:** 2015-03-23

**Authors:** Guo-ping Xie, Nan Jiang, Chang-xiang Liang, Jian-chun Zeng, Zhi-yuan Chen, Qian Xu, Rui-zhen Qi, Yi-rong Chen, Bin Yu

**Affiliations:** 1 Department of Orthopaedics and Traumatology, Nanfang Hospital, Southern Medical University, Guangzhou, Guangdong, PR China; 2 Key laboratory of Bone and Cartilage Regenerative Medicine of Guangdong Province, Nanfang Hospital, Southern Medical University, Guangzhou, Guangdong, PR China; 3 Department of Orthopaedics, Guangdong General Hospital, Guangzhou, Guangdong, PR China; 4 Department of Third Orthopaedics, The First Affiliated Hospital, Guangzhou University of Traditional Chinese Medicine, Guangzhou, Guangdong, PR China; 5 Department of Overseas Chinese Patients, General Hospital of Guangzhou Military Command, Guangzhou, Guangdong, PR China; 6 Department of Health Statistics, Guangzhou University of Traditional Chinese Medicine, Guangzhou, Guangdong, PR China; University of Texas Health Science Center at Houston, UNITED STATES

## Abstract

**Purpose:**

To review clinical characteristics of pigmented villonodular synovitis (PVNS) in China.

**Methods:**

Electronic medical records (EMR) of four Chinese institutes were queried for patients with histologically proven PVNS between January 2005 and February 2014. Their data were collected including gender, age at diagnosis, clinical presentation, affected site, symptom duration, comorbidities, treatment strategy, recurrence and routine laboratories.

**Results:**

A total of 237 patients with biopsy-proven PVNS were investigated. The gender ratio was 1.35 for a female predominance (101 males and 136 females). The average age was 36 years (range, 2 to 83 years). The median delay from initial clinical symptom to diagnosis was 18 months. Main affected areas were the knee (73.84%) and the hip (18.14%). Forty patients had a clear history of joint trauma. Six patients were concurrently diagnosed with PVNS and avascular necrosis (AVN). Five patients suffered from PVNS following implantation of orthopaedic devices including artificial prosthesis, plate and wire. One hundred and twenty-nine patients underwent arthroscopic synovectomy and 108 open synovectomy. Altogether 48 patients (26 males and 22 females) had recurrence of disease. The relapse rate was 24% (knee) and 6.98% (hip), 20.93% (open surgery) and 19.44% (arthroscopy), respectively. Erythrocyte sedimentation rate (ESR) and C-reactive protein (CRP) rate were elevated in 45.83% and 38.41% of the patients respectively.

**Conclusions:**

To our knowledge, this study is the largest sample size of PVNS patients reported as well as the largest sample of PVNS with concurrent AVN reported to date. Our outcomes suggest that PVNS shows a female predominance, occurs mostly between 20–40 years and favors the knee and hip. Recurrence is frequent, particularly in the knee. Serum ESR and CRP may be elevated in some patients. Additionally, the present study supports the theory of an association between PVNS and orthopedic surgery, which is not limited to joint replacement.

## Introduction

Pigmented villonodular synovitis (PVNS), first coined by Jaffe et al. [1] in 1941, is a rare, benign, but potentially locally aggressive and recurrent disease. It is characterized by synovial hyperplasia and pigment deposition (hemosiderin) inside the joints, tendon sheaths and bursae. Evidence shows that it mainly affects large joints such as the knee and the hip [2,3], and is extremely rare in the temporomandibular joint [4] and spine [5]. It mainly occurs between 20–40 years, though it is also found in children and the elderly. The annual incidence of PVNS was estimated to be 1.8/1000000 in the USA [6].

Although PVNS is believed to be a benign proliferation of the synovium, its etiology is unclear to date. Some [7,8] think it is a chronic inflammatory disease while others [9,10,11] believe it is a tumor-like illness which rarely metastasizes [12,13]. The association between a history of trauma and PVNS is unclear. Mayer et al. [6] reported that 53% of the PVNS patients had a positive history of trauma, while only 25% of such patients were reported by Sharma et al. [14].

PVNS clinical reports vary based on the country of origin, regions, ethnic groups and races [6]. With respect to its gender ratio, a male predominance was reported in United States [6] and United Kingdom [14,15], but a female predilection in France [16] and Portugal [17]. In China, Ma et al. [3] reported the gender ratio (female/male) was 1.78.

Currently, studies on PVNS mainly focus on its radiological and pathological characteristics and surgical outcomes. Little effort has been made to address its clinical characteristics. Therefore, we conducted this multicenter investigation of the clinical profile of the disease in South China. We hoped that our findings will enhance our understanding of the disease and provide some clues that might help answer questions about the etiology and diagnosis of the disease, as we were able to assemble the largest sample of PVNS patients reported to date.

## Materials and Methods

### Study design

This study was a systematic multicenter retrospective analysis of the clinical characteristics of PVNS.

### Data sources and patients selection

Clinical data of all the patients with biopsy-proven PVNS between January 2005 and February 2014 were obtained from the electronic medical records (EMR) of four hospitals in south China: Nanfang Hospital, Guangdong General Hospital, The First Affiliated Hospital to Guangzhou University of Traditional Chinese Medicine and General Hospital of Guangzhou Military Command. Index term ‘‘pigmented villonodular synovitis” was set for search. The initial patients’ records were reviewed for eligible assessment.

### Inclusion and exclusion criteria

Eligible patients were those with definite histological PVNS diagnosis with available clinical data regarding gender, age at diagnosis, initial clinical symptoms, affected site, symptom duration, treatment strategy, incidence of recurrence, and preoperative serum inflammatory markers including white blood cell count (WBC), erythrocyte sedimentation rate (ESR) and C-reactive protein (CRP). Patients that were initially suspected to have PVNS but ruled out by final pathological diagnosis and those who had insufficient clinical data were excluded from this study.

This study was approved by medical ethics committee of Nanfang Hospital. All clinical investigation was conducted according to the principles expressed in the Declaration of Helsinki. Written consents from the patients were waived, but the patients’ records were anonymized and deidentified prior to analysis.

### Statistical analysis

Statistical analysis was performed using the SPSS 19.0 software. Measurement data were described by means and standard deviations (SD) if normally distributed and medians along with interquartile ranges (IQR) if not normally distributed. Dichotomous variables were expressed as percentages. T-test was performed to evaluate the differences of continuous variables. Chi-square test was used to assess the differences between dichotomous variables. Comparisons of anatomical distributions and initial clinical symptoms were performed by Monte Carlo’s exact test. *P* value of ≤0.05 was considered statistically significant.

## Results

### Patient identification and gender ratio

**Fig 1:**
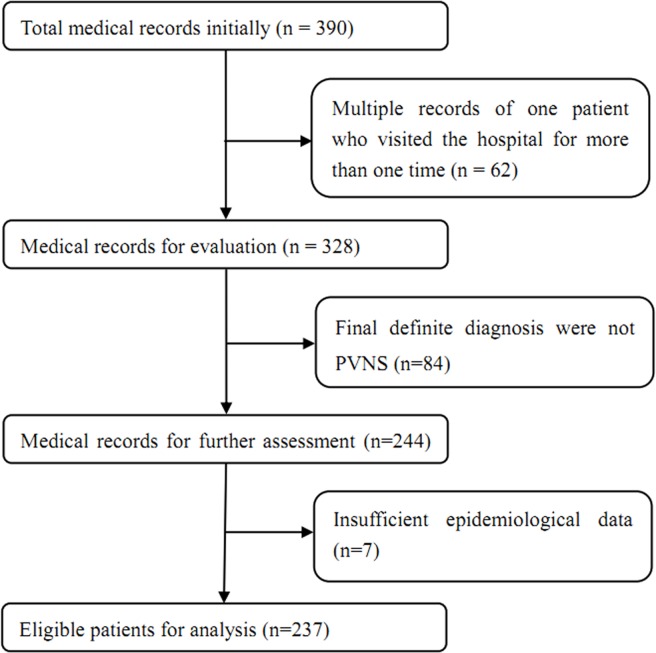
Flow chart of eligibility selection.

A total of 390 patients were identified initially. After reviewing the records, we finally included 237 eligible patients for the present study. The identification and inclusion process was illustrated in Fig. 1. Of the eligible 237 patients, 136 were female and 101 male, giving a gender ratio (female/male) of 1.35 (Table 1). Stratified analysis of the gender ratio according to the affected site (knee and hip) resulted in 1.24 for the knee and 1.53 for the hip (Table 1).

**Table 1:** Clinical and laboratory features of studied PVNS patients.

	Total	Male	Female	Knee	Hip
**Number** (%)	237	101 (42.62)	136 (57.38)	175 (73.84)	43 (18.14)
**Mean age at diagnosis** (mean±SD)(Y)	35.52±15.31	34.65±15.24	36.17±15.40	35.70±16.12	32.07±10.12
**Gender ratio** (females/males)	1.35 (136/101)	-	-	1.24 (97/78)	1.53 (26/17)
**Laterality** [no.(%)]					
*Left*	111(46.84)	47(19.84)	64(27.00)	78(32.91)	24(10.13)
*Right*	119(50.21)	50(21.10)	69(29.11)	91(38.40)	18(7.59)
*Bilateral*	7(2.95)	4(1.69)	3(1.26)	6(2.53)	1(0.42)
**Median interval from symptoms onset to diagnosis** (range) (M)	18(0.2–600)	19.5 (0.2–360)	18(0.5–600)	12(0.2–600)	24(1–240)
**Initial clinical symptom** [no.(%)]					
*Pain*	106(44.73)	43(18.14)	63(26.58)	60(25.32)	40(16.88)
*Swelling*	53(22.36)	22(9.28)	31(13.08)	66(27.85)	0(0.00)
*Swelling and pain*	70(29.54)	33(13.92)	37(15.61)	44(18.57)	0(0.00)
*Other*	8(3.37)	3(1.27)	5(2.11)	5(2.11)	3(1.27)
**Comorbidities** [no.(%)]					
*Avascular necrosis*	6(2.53)	2(0.84)	4(1.69)	1(0.42)	5(2.11)
*Orthopedic intervention*	5(2.11)	4(1.69)	1(0.42)	3(1.27)	1(0.42)
**Trauma history**	40(16.88)	18(7.59)	22(9.28)	31(13.08)	6(2.53)
**Treatment strategies** [no.(%)]					
*Arthroscopy*	129(54.43)	56(23.63)	73(30.80)	118(49.79)	6(2.53)
*Open surgery*	108(45.57)	45(18.99)	63(26.58)	57(24.05)	37(15.61)
**Recurrence** (events/total) [no.(%)]	48/237 (20.25)	26/101 (25.74)	22/136 (16.18)	42/175 (24.00)	3/43 (6.98)*
**Median recurrence time** (range) (M)	12(1–144)	12(1–108)	16.5(1–144)	12(1–120)	108(24–144)
**Serum inflammatory marker**					
*WBC (mean ± SD) (×10* ^*9*^/*L)*	7.04±2.05	7.59±2.18	6.66±1.87	6.97±2.18	7.23±1.83
*ESR (median*, *IQR)(mm/h)*	13.5 (7.00, 30.00)	11.00 (4.00, 28.00)	14.00 (7.75, 30.25)	13.00 (7.00, 33.00)	10.00 (6.00, 20.00)
*CRP (median*, *IQR)(mg/L)*	3.06 (1.20, 8.96)	3.64 (1.72, 23.35)	2.38 (1.00, 6.71)	2.76 (1.00, 14.33)	3.23 (1.48, 7.34)

WBC = white blood cell count, ESR = erythrocyte sedimentation rate, CRP = C-reactive protein, IQR = interquartile range, SD = standard deviation, M = month, Y = year.

* *p*≤0.05

### Age and symptom duration at the time of diagnosis

**Fig 2:**
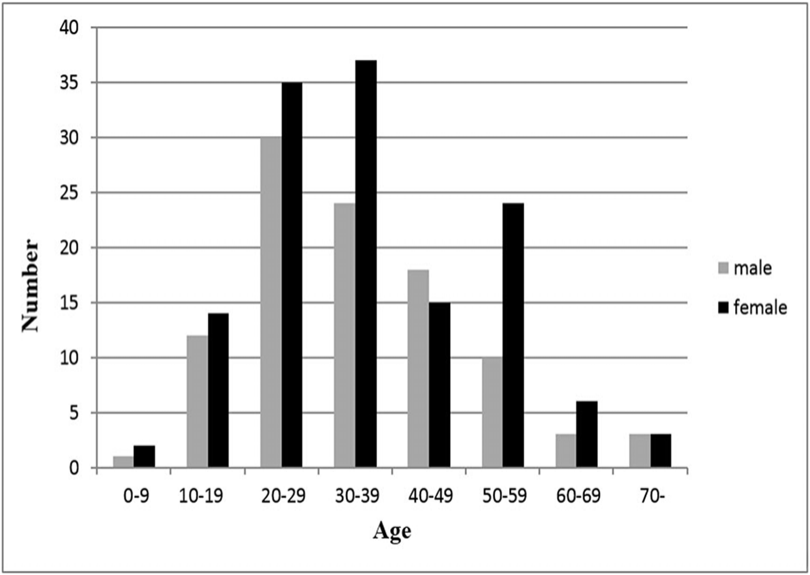
Age distribution of 237 PVNS patients by gender.

**Fig 3:**
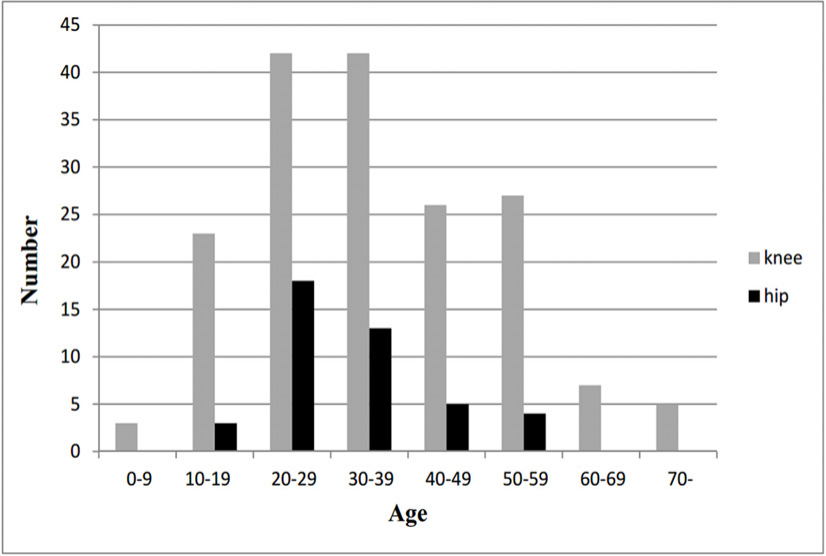
Age distribution of 237 PVNS patients by knee and hip.

The overall average age at diagnosis was 36 years (range, 2 to 83 years), no significant difference was identified between the two genders (*p* = 0.452). As shown in Figs. 2 and 3, the disease mainly occurred between 20–59 years, with the second and third decades being the peak. The mean age at diagnosis did not differ significantly between the hip and the knee patients (*p* = 0.068) (Table 1). The median delay from initial clinical symptoms to diagnosis was 18 months (range, 0.2 to 360 months). Although it was somewhat longer in males than in females, there was no statistically significant difference (*p* = 0.505).

### Affected site and laterality

**Fig 4:**
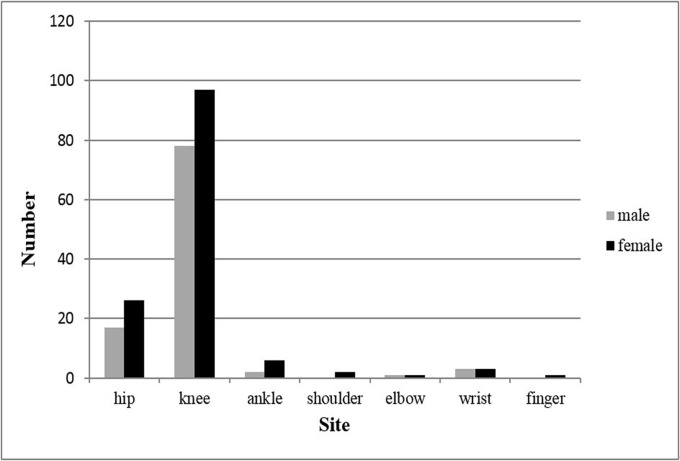
Anatomical distribution of 237 PVNS patients by gender.

In current study, no cases of metastatic or “tumor-like” PVNS were seen in this sample. As shown in Table 1, the knee (175 cases, 73.84%) was the most frequent site affected, followed by hip (43 cases, 18.14%), ankle (8 cases), wrist (6 cases), shoulder (2 cases), elbow (2 cases) and finger (1 case). Anatomical distributions of the disease did not differed significantly between genders (*p* = 0.78) (Fig. 4). PVNS occurred on the left side in 111 patients, on the right side in 119 and on the bilaterally in 7.

### Initial clinical presentation

Initial clinical symptoms were pain and/or swelling in most cases. Six patients reported other symptoms such as fatigue, limited range of motion and mechanical “locking phenomenon” (sensation of joint locking during movement). No symptoms showed in two patients whose PVNS was found incidentally during surgery for other diseases. A clear history of joint trauma (including sprain, falling, crush and traffic injury) was reported in 40 patients (18 males and 22 females), of whom 31 had the knee PVNS. The other patients (197 patients) had no trauma history (Table 1).

### Comorbidities

#### Concomitant PVNS and AVN

Six patients were histologically confirmed to have PVNS along with femoral head avascular necrosis. They were diagnosed at an average age of 33 years. AVN was diagnosed concurrently in 5 cases all of which occurred in the hip with a radiological severity ranging from type II to type IV by Ficat grading system and histologically proven. One case with histopathological diagnosis of right knee PVNS had compatible AVN of the ipsilateral femoral head. The detailed information for these patients is shown in Table 2. Clinical reports of PVNS cases with AVN are still limited [32]. To our knowledge, this is the largest sample size of PVNS with AVN reported currently. Similarly, our findings supported the statement by Cotten et al. [32] that PVNS and AVN are compatible. However, we are still unclear about the relationship between PVNS and AVN.

**Table 2:** Clinical characteristics of PVNS patients with AVN.

Case no.	Gender	Age (Y)	Laterality	Ficat type	Alcohol abuse	Glucocorticoid history	Trauma history	Initial symptom	WBC (×10^9^/L)	ESR (mm/h)	CRP (mg/L)	Affected site	Same joint
Case 1	Male	16	Right	II	No	No	No	Swelling, pain	9.17	16	3.77	Knee	No
Case 2	Male	28	Left	IV	No	No	No	Pain	8.13	4	0.17	Hip	Yes
Case 3	Female	31	Left	II	No	No	No	Pain	7.23	6	1.75	Hip	Yes
Case 4	Female	35	Right	III	No	No	No	Pain	7.59	70	NA	Hip	Yes
Case 5	Female	35	Bilateral	II	No	No	No	Pain	5.15	50	7.91	Hip	Yes
Case 6	Female	55	Left	IV	No	No	No	Pain	7.10	4	0.32	Hip	Yes

WBC = white blood cell count, ESR = erythrocyte sedimentation rate, CRP = C-reactive protein.

NA = not available, Y = year.

The cut-off values for serum WBC, ESR and CRP are 9.5 × 10^9^/L, 15 mm / h, 5 mg / L, respectively.

#### PVNS in patients with orthopedic surgery history

In this study, five patients with PVNS had a definite history of orthopedic surgery in the joint affected by PVNS. The affected joints were the knee (3 cases), the hip (1 case) and the wrist (1 case). The surgical procedures included single femoral condylar replacement (1 case), total hip arthroplasty (1 case), plate fixation for bone fracture (2 cases) and wire fixation for reconstruction of anterior cruciate ligament (1 case). The detailed information for these patients was revealed in Table 3.

**Table 3:** Clinical characteristics of PVNS patients diagnosed after orthopedic interventions

Case no.	Gender	Age (Y)	Site	Primary indication	intervention	Materials	Time interval * (M)
Case 1	Male	36	Right knee	Fracture	Plate fixation	Metal	11
Case 2	Male	38	Left knee	Cruciate ligament rupture	Wire fixation	Metal	120
Case 3	Female	55	Left hip	Osteoarthritis	Total hip arthroplasty	Polyethylene	66
Case 4	Male	60	Left wrist	Fracture	Plate fixation	Metal	12
Case 5	Male	75	Right knee	Osteoarthritis	Unicondylar arthroplasty	Metal	60

M = month, Y = year.

*Time interval = The interval between the surgery for the primary disease and the occurrence of PVNS.

### Preoperative WBC, CRP and ESR

Preoperative WBC, ESR and CRP were respectively performed for 222, 144 and 138 patients prior to surgery (1–5 days, average 2.44 days) (Table 4). The ESR was elevated in 45.8% of patients tested with a median of 13.5 mm/h (cut-off value 15 mm/h), the CRP was elevated in 38.41% of patients tested with a median of 3.06 mg/L (cut-off value 5 mg/L) and finally the WBC were elevated preoperatively in 38.41% of patients tested with a mean of 7.04×10^9^/L (cut off value 9.50×10^9^/L) (Table 1, 4). As revealed in Table 4, no significant differences were identified regarding the incidence of recurrence between the patients with elevated or normal serum inflammatory markers except for a slightly higher recurrence rate in the group with knee PVNS and elevated CRP.

**Table 4:** Clinical and laboratory presentation of recurring PVNS cases

	Total	Knee	Hip
**Trauma history**			
*Prior trauma*	4/40	4/31	0/6
*No trauma*	44/197	38/144	3/37
**Treatment strategy**			
*Arthroscopy*	27/129	26/118	0/6
*Open surgery*	21/108	16/57	3/37
*Synovectomy*	20/86	16/44	2/28
*Arthroplasty*	1/22	0/13*	1/9
**Serum inflammatory marker**			
WBC			
*Normal value*	43/202	37/146	3/38
*Elevated value*	4/20	4/16	0/3
ESR			
*Normal value*	13/78	12/49	1/24
*Elevated value*	15/66	13/46	2/13
CRP			
*Normal value*	10/85	8/54	2/24
*Elevated value*	13/53	13/38*	0/12

WBC = white blood cell count, ESR = erythrocyte sedimentation rate, CRP = C-reactive protein.

All the data are expressed in events/total.

* *p*≤ 0.05.

### Imaging features of PVNS

Plain radiographs usually show abnormal signals in the affected joint of PVNS patients. However, the changes in radiographs in some PVNS patients are untypical, MRI test should be performed for further evaluation. The typical histopathological features of PVNS are synovial hypertrophy with villositary structure and local hemosiderin hyperpigmentation. Here we listed two cases to illustrate imaging features of PVNS.

**Fig 5:**
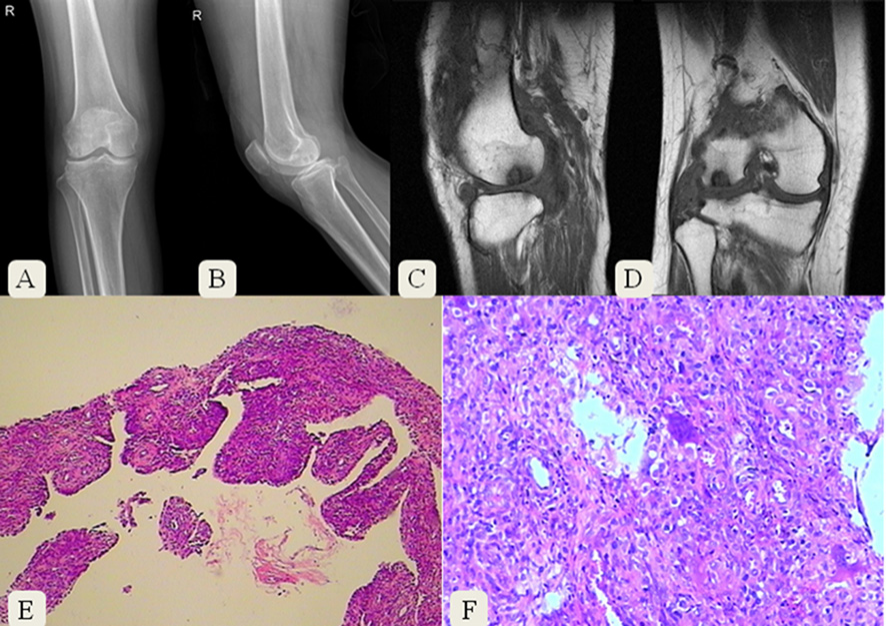
Radiographs, MRI and histopathological images of a 56-year-old female diagnosed with right knee PVNS.

A 56-year-old female was diagnosed of right knee PVNS. Anteroposterior and lateral radiographs showed two oval low density regions in the condyle of the right femur (Fig. 5A-B). MRI clearly revealed abnormal low signals in the lateral femoral condyle with synovium thickening around (Fig. 5C-D). Histopathologic images showed synovial hypertrophy, multinucleated giant cells and local hyperpigmentation of hemosiderin (Fig. 5 E-F).

**Fig 6:**
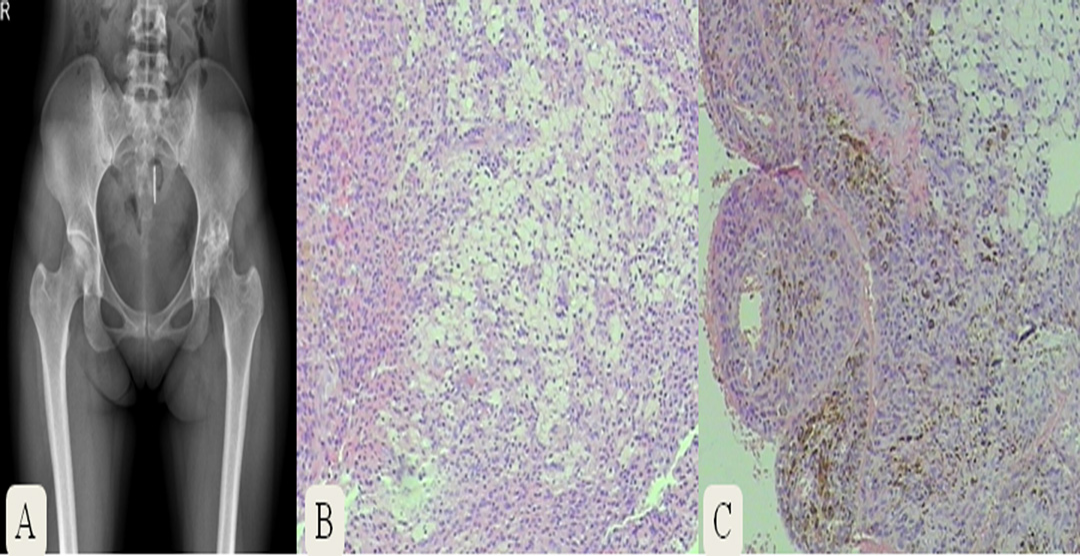
Radiograph and histopathological images of a 31-year-old female diagnosed with left hip PVNS.

A 31-year-old female was diagnosed of left hip PVNS. The plain radiograph showed multiple cystic lesions with joint space narrowing in the left hip (Fig. 6A). Histopathologic images revealed nodular hyperplasia of the synovium, foamy cells and hemosiderin hyperpigmentation (Fig. 6B-C).

### Treatment strategy and recurrence

Of the 237 patients, 129 underwent arthroscopic synovectomy and 108 open synovectomy (including 86 simple synovectomy and 22 arthroplasty) (Tables 1 and 4). Altogether 48 patients had recurrence of the disease. Patients with knee PVNS had a significantly higher recurrence ratio (42/175, 24.0%) than those with hip PVNS (3/43, 6.98%) (*p* = 0.01) (Table 1). But no significant gender difference was identified in the recurrence ratio (*p* = 0.07). The recurrence ratio in patients with trauma history (4/40, 10%) was significantly lower than those without trauma history (44/197, 22.34%), but this difference did not reach statistical significance (*p* = 0.09) (Table 4). In addition, no significant recurrence difference was identified between patients that were treated with open versus arthroscopic surgery. (*p* = 0.78) (Table 4).

## Discussion

This multicenter retrospective study, based on a large sample, reveals that PVNS had a female predilection in Chinese population, peaked between 20–40 years, and favored the knee. No significant differences were identified between the two treatment strategies or between genders regarding the recurrence rate of knee PVNS, which was approximate 20% after the first surgery. We also observed that a large proportion of patients had elevated inflammatory markers.

With respect to the gender ratio, our result was similar to another Chinese report [3], and to those reported in France (1.36) [16] and Portugal (1.15) [17]. However, a male predilection was reported in United States (0.53) [6], United Kingdom (0.6) [14] and Netherlands (0.81) [18]. Just as Myers et al. [6] and Ottaviani et al. [16] indicated, race and regional differences might affect the gender ratio of PVNS.

The present study found that the average age at diagnosis was around 35 years though PVNS occurred in a two-year-old child and an 83-year-old man. These results are similar to some studies [14,16,18], but others [3,19] have observed an older age range (44–59 years). The disease was diagnosed usually between 20–59 years, third and fourth decades in particular. This is in agreement with previous studies [18,20,21]. We found no significant differences for age at diagnosis between genders or affected sites.

Our result showed that the median interval from appearance of initial clinical symptoms to a definite diagnosis was 18 months, similar to a previous report [19] of 20 months. However, the average durations were quite different in another three studies, with outcomes ranged from 5 months [22], 35 months [16] to 54 months [3]. This was probably attributed to the nonspecific clinical signs of PVNS and coexistence of other diseases [23,24]. In addition, male patients had a significantly longer median delay than females in our study.

With respect to site distribution of PVNS, some studies [11,16,25] reported that the top two frequent locations of the disease were the knee and the ankle, but other studies [3,26] and the present one found the two were the knee and the hip. One thing is certain that the knee is the most affected joint by the disease. Our study revealed no significant difference in incidence of the disease between left and right sides of the body.

Most of the previous [3,16,17,27] studies reported the clinical symptoms of the disease were pain and aggravated swelling, which were also the most frequent initial symptoms found in our study. Nonetheless, we found that the symptoms differed in different locations. The most frequent symptom for knee PVNS was pain with swelling, while in the hip joint pain was the top symptom. Patients with hip PVNS usually complained less of swelling not because of absence of swelling but because they did not perceive the symptom which might have been covered by the deep anatomic structure and stronger muscles around the hip joint. Similar to those with other osteoarticular disorders, patients with PVNS usually do not perceive obvious symptoms at an early stage offering few specific predictors at disease onset [6].

Controversy still surrounds the features of PVNS. It was once regarded as an inflammatory disease [1,8,28], but in recent years more and more literature [9,13,29,30] reported its tumor-like characteristics. Likewise, the etiology of PVNS is still unclear. It was reported that the disease was associated with lipometabolism and trauma [6,31]. Therefore, we investigated the medical history and accompanied diseases. We found 16.88% of the patients had a definite trauma history but only a few patients had comorbidities, including popliteal cyst and vascular tumor. The present study failed to confirm whether the above accompanied diseases were risk factors of PVNS.

PVNS with concomitant AVN has been rarely reported [32], but the present study identified a total of six cases of this kind, five of which had PVNS and AVN in the same joint (hip). However, no risk factors (e.g. alcohol, glucocorticoids, trauma) accounting for AVN were identified. With regard to the potential biological reasons for the coexistence of PVNS and AVN, we inferred that PVNS may result in or accelerate the process of AVN. Our hypothesis is that the synovial hyperplasia may reduce the blood flow to the periarticular bone [32] and the inflammatory process of PVNS may lead to the necrosis of the femoral head.

A few studies report an association between orthopedic interventions (specifically arthroplasty) and PVNS. We observed that five patients had orthopedic implants before occurrence of PVNS. There were few case reports on PVNS after orthopaedic implants, and reported cases only occurred after arthroplasty [33,34,35]. Here we reported three cases suffered from PVNS following internal fixations. The clinical phenomenon implies that PVNS may be a complication of orthopedic surgery. As for possible reasons, Ballard et al. [36] indicated that the components of orthopedic implants (including metal and polyethylene) may be acted as inflammatory factors resulting in the hyperplasia of synovial membrane.

Until now knowledge regarding preoperative WBC, ESR and CRP levels in PVNS patients is still limited. Several studies [6,32,37] report that most PVNS patients had their values of WBC and ESR in the normal range. However, we found significantly higher positive ratios of preoperative serum ESR (45.83%, 66 of 144 cases) and CRP (38.41%, 53 of 138 cases), followed by WBC (9.01%, 20 of 222 cases). This implies that PVNS may have an effect on serum inflammatory markers and should be regarded as an active disorder. Nonetheless, it should be noted that ESR and CRP are not specific indicators of PVNS. On one hand, diseases like tuberculosis, tumor, systematic or local infections can also result in the elevation of ESR and CRP. On the other hand, not all PVNS patients had elevated values of ESR and CRP. Due to the controversy between the present study and previous studies regarding serum inflammatory markers, further investigations are necessary with a larger sample size.

The main management strategy of PVNS is synovectomy, largely achieved by arthroscopy and open surgery (including arthroplasty) [38,39]. It was performed in all the 237 patients in our study, 48 of whom experienced recurrence postoperatively. The recurrence ratio in male patients was higher than that in females but was not statistically significant (*p* = 0.07). In the patients with recurrence in the present study, the knee PVNS accounted for the largest proportion (24%), consistent with the finding by Ottaviani et al. [16]. Although the occurrence rate showed no significant difference between the patients with and without a trauma history, it was lower in the former ones (10%) than in the latter ones (22.34%). Therefore, it is still unclear whether trauma may play a role in the recurrence of PVNS. Likewise, disputes still exist regarding the association between recurrence and treatment. Various recurrence rates with different treatment strategies and follow-up durations were described, ranging from 8% to 46%, in the literature [20,40,41]. Some studies [3,39] reported an increased recurrence rate following open surgery while others [27,42] indicated a lower recurrence rate following open surgery compared with arthroscopy. However, we found similar rates between the two treatment strategies. In addition, we noticed that the patients undergoing arthroplasty suffered from the lowest recurrence susceptibility. The contradictory reports about the association between recurrence and treatment may be explained by the fact that many factors other than the treatment strategies of PVNS might have affect the recurrence rate, such as severity and location of the primary lesion and surgeons’ expertise.

There are several limitations in our study. Firstly, although the present study has reported the largest collection of PVNS cases up until now, clinical data were just from four hospitals in South China. Several significant clinical indicators were not reported (e.g. the incidence of the disease) because our sample size was still limited and corresponding denominators were unavailable. Secondly, due to the retrospective and non-case-control nature of the present study, biases cannot have been avoided, such as recall bias, and risk factors of the disease were not explored. Finally, the recurrence rate was calculated just based on EMR rather than complete follow-up information, which might have affected the accuracy of this indicator. In the present study, follow-up data of patients were incomplete, which was mainly due to the high mobility of patients in China. Therefore, the present study inevitably possesses biases which may have affected the outcomes and we did not sort the disease manifestations based on pathological classification though all cases were diagnosed by histological evaluations. Moreover, inconsistency in follow-up time may have affected evaluation of the curative effect and the recurrence rate.

## Conclusions

In summary, to our knowledge, this is the largest sample of PVNS and PVNS with concurrent AVN reported to date. Our results show that PVNS is predominant in female, frequently occurs between 20 to 40 years and favors the knee joint, which is also the most likely location of recurrent PVNS regardless of treatment strategies and genders. In addition, we find serum ESR and CRP may be elevated in some patients. Moreover, our reported cases support the theory of an association between PVNS and orthopedic surgery, which is not limited to joint replacement.

## Supporting Information

S1 Datafile All data of the PVNS patients. (XLSX)
